# Hematological Toxicity in Mice after High Activity Injections of ^177^Lu-PSMA-617

**DOI:** 10.3390/pharmaceutics14040731

**Published:** 2022-03-28

**Authors:** Amanda Kristiansson, Oskar Vilhelmsson Timmermand, Mohamed Altai, Joanna Strand, Sven-Erik Strand, Bo Åkerström, Anders Örbom

**Affiliations:** 1 Department of Clinical Sciences Lund, Oncology, Lund University, 222 42 Lund, Sweden; oskar.vilhelmsson_timmermand@med.lu.se (O.V.T.); mohamed.altai@med.lu.se (M.A.); joanna.strand@med.lu.se (J.S.); sven-erik.strand@med.lu.se (S.-E.S.); anders.orbom@med.lu.se (A.Ö.); 2 Department of Hematology, Oncology, Radiation Physics, Skåne University Hospital, Lund University, 222 43 Lund, Sweden; 3 Department of Clinical Sciences Lund, Medical Radiation Physics, Lund University, 221 85 Lund, Sweden; 4 Department of Clinical Sciences Lund, Section for Infection Medicine, Lund University, 221 84 Lund, Sweden; bo.akerstrom@med.lu.se

**Keywords:** radioligand therapy, ^177^Lu-PSMA-617, prostate cancer, mouse model, hematotoxicity

## Abstract

Prostate cancer (PC) is one of the most common malignancies affecting men, with poor prognosis after progression to metastatic castration-resistant prostate cancer (mCRPC). Radioligand therapy (RLT) targeting the overexpressed PSMA on PC cells, with, e.g., ^177^Lu-PSMA-617, has been effective in reducing tumor burden and prolonging survival in mCRPC. However, it is not a curative method with kidney and bone marrow toxicity limiting the activity given to patients. Previous preclinical models have reported transient hematotoxicity for up to 120 MBq. This activity may still be too low to investigate the effect on renal function since it corresponds to an absorbed dose below 10 Gy, whereas the kidneys in a clinical setting usually receive an absorbed dose more than double. Here we investigated the hematotoxicity and recovery after administered activities of 120, 160, and 200 MBq in a ^177^Lu-PSMA-617 BALB/cAnNRj mouse model. The animals had an initial drop in white blood cells (WBC) starting 4 days post injection, which recovered after 21 days. The effect on red blood cells (RBC) and platelets was detected later; 17 days post-injection levels decreased compared to the control group. The reduction was restored again 32 days post injection. No correlation between injected activity and hematotoxicity was found. Our results suggest that activities up to 200 MBq of ^177^Lu-PSMA-617 give transient hematotoxicity from which animals recover within a month and no radiation-related deaths. Injecting these high activities could allow animal studies with increased clinical relevance when studying renal toxicity in animal models.

## 1. Introduction

Prostate cancer (PC) is one of the most frequent malignancies affecting men. In locally advanced and metastatic disease, androgen deprivation therapy (ADT) is often the first-line treatment with initially great response rates. However, androgen ablation does not cure prostate cancer, and eventual disease progression to androgen-independent or castration-resistant prostate cancer (CRPC) is common [1]. At this stage, many patients have formed metastases (mCRPC), e.g., in the skeleton or liver, lowering the failure-free survival to under a year as reported in the PREVAIL and STAMPEDE trials [2,3].

A novel treatment modality that can be used to alleviate symptoms of mCRPC is radioligand targeting of prostate-specific membrane antigen (PSMA). PSMA is a transmembrane protein that is overexpressed in PC, with moderate expression in some normal tissues such as the kidney, salivary glands, and proximal small intestine [4]. PSMA is both a predictor of disseminated disease and a therapy target since PSMA expression increases with the severity of the disease (e.g., tumor grade, metastatic disease, reoccurrence, and androgen-independence) [5]. Treatment with PSMA radioligand therapy (RLT), with, e.g., ^177^Lu-PSMA-617 or ^177^Lu-PSMA-I&T, is not curative but has been effective in reducing tumor burden and increasing life expectancy in patients with mCRPC [6,7,8]. However, studies have mostly been performed on terminally ill patients, and PSMA-RLT is not a curative method today.

The limiting factor in RLT is to keep the absorbed dose to normal tissue low while delivering high absorbed doses to cancer cells. Two of the most important dose-limiting tissues in RLT are the bone marrow and the kidneys. Hematologic adverse events have been reported more frequently than diminished renal function with ^177^Lu-PSMA-617. For example, Rahbar et al. reported grade 3–4 hematotoxicity in 12% of patients (3% leukopenia, 10% anemia, and 4% thrombocytopenia) in a multicenter trial, and Groener et al. reported similar numbers (9.3%; 3.6% leukopenia, 7.1% anemia, and 4.3% thrombocytopenia) [9,10]. There are several risk factors for hematotoxicity, e.g., patients with high occurrence of bone metastases, previous chemotherapy with taxane, or pretreatment grade 2 cytopenia [10].

Relevant animal models are needed to evaluate efficacy and toxicity to enable optimization of RLT alone or in combination with other therapies, for example, chemotherapy, inhibitors of DNA damage repair, or radiosensitizers (reviewed in [11]). Moreover, therapy combinations, e.g., infusion of amino acids, PSMA inhibitors, and α_1_-microglobulin (A1M), may be aimed at reducing radiation-related side-effects [12,13,14,15]. Our group has focused on the use of A1M, an antioxidant with radical scavenging, reductase, and heme-binding abilities (reviewed in [16]). In the liver, there is a continuous production of A1M, which is released into the blood circulation, with clearance occurring in the kidneys. A1M has been shown to reduce kidney injury in peptide receptor radionuclide therapy with ^177^Lu-DOTATATE and RLT with ^177^Lu-PSMA-617 in mice without interfering with tumor treatment [12,17,18].

For optimization of radionuclide therapies, the development of animal model systems of these treatments is necessary. To accomplish relevant models, injecting activities yielding similar absorbed doses or biological effects as seen in humans is necessary. Fendler et al. showed that animals injected with up to 120 MBq of ^177^Lu-PSMA-617 had fully recovered white blood cells (WBC), red blood cells (RBCs), and platelet counts after four weeks [19]. It is of importance to understand the hematotoxicity associated with ^177^Lu-PSMA-617, as cytopenia is more common than kidney toxicity in patients treated with ^177^Lu-PSMA-617 and could hinder the potential of administering higher activities. Further, a suitable mouse model of kidney toxicity following ^177^Lu-PSMA-617 treatment would require total injected activities of more than 400 MBq (about 29 Gy absorbed dose to kidneys). It is, however, unclear whether it is possible to achieve such high absorbed doses to the kidneys of mice without severe hematotoxicity as studies, including our own, have injected between 100 and 120 MBq of ^177^Lu-PSMA-617. The aim of this study was to investigate the hematotoxicity, tolerance, and recovery after administration of activities up to 200 MBq in a ^177^Lu-PSMA-617 mouse model.

## 2. Materials and Methods

### 2.1. Radiosynthesis

In short, following a previously published protocol [12,19,20], 100 µL of sterile sodium acetate solution (0.4 M, pH 5.5) and 1.25 µL ascorbic acid solution (20% *w*/*w*) were added to ca 30–40 µL (1.25 GBq in total) of non-carrier added Lutetium-177 (ITM Isotope Technologies Munich, Garching, Germany). To this solution, 20 nmol (volume circa 2 µL) of PSMA-617 (MedChemExpress, Monmouth Junction, NJ, USA) were added and labeled by incubating on a shaker at 95 °C for 15 min after which the reaction was terminated by cooling to room temperature. At 0 and 15 min, respectively, 1 µL of the solution was added to an instant thin layer chromatography (iTLC) strip. Sodium citrate solution (0.2 M, pH 2) was used as mobile phase, and the percentage of free ^177^Lu (which migrates with the solvent front) was determined by analyzing the iTLC strips with a phosphor imager system (Cyclone Plus Phosphor Imager, PerkinElmer, Inc., Waltham, MA, USA). A sterile 0.9% sodium chloride solution was added, and the radioligand diluted (1:3 or 1:7), a sample for iTLC taken, and pH tested before injections in mice. ^177^Lu-PSMA was labeled at a specific activity of 62 MBq/nmol (judged most effective for therapy by Fendler et al. [19]), and the radiochemical purity of the radioligand was >99%.

### 2.2. In Vivo Studies

All experiments were conducted in accordance with the directions given by the regional ethical committee for animal trials (Dnr: 04350-2020 with the addition Dnr 5.8.18-07300/2021). BALB/cAnNRj mice (Janvier Labs, Le Genest-Saint-Isle, France) were injected with 120 (107–124, *n* = 8), 160 (147–169, *n* = 13) or 200 (188–201, *n* = 6) MBq of ^177^Lu-PSMA-617 and one group was injected with PBS (vehicle, *n* = 10). Injected ligand amount per mouse was 1.9, 2.6, and 3.2 nmol in the three groups, increasing with injected activity. The differences in group size are due to unexpectedly high activities remaining in the syringe post injection. The volume of radioligand injected per mouse was up to 100 µL (200 MBq). Animals were weighed continuously throughout the study and sacrificed after 32 days.

### 2.3. Blood Cell and Platelet Count

Blood was continuously sampled (4, 13, 17, 21, 25, and 32 days) after injection of ^177^Lu-PSMA-617. Mice were sampled every other time point (one mouse was sampled on three occasions). Samples (20 µL) were collected from the tail vein from awake, immobilized mice by piercing the vein with a needle (27 G) and collecting blood in a K2EDTA-coated plastic micropipette (Boule Medical, Stockholm, Sweden). If not a sufficient amount of blood could be taken in three tries or clogging of capillary occurred, the animal was excluded from that time point. Total white blood cell counts (WBC, 10^9^/L), lymphocytes (LYM, 10^9^/L), monocytes (MONO, 10^9^/L), granulocytes (GRAN, 10^9^/L), hemoglobin (HBG, g/dl), hematocrit (HCT, %), red blood cell counts (RBC, 10^12^/L), mean cell volume (MCV, femtoliter = fl) and platelets (PLT, 10^9^/l) were measured in an Exigo Veterinary (Exigo Vet) Hematology Analyzer (Boule Medical, Stockholm, Sweden).

### 2.4. Statistical Analysis

Data are presented as mean ± SEM if not specified otherwise in figure legends. Statistical significance was calculated with a one-way ANOVA test corrected for multiple comparisons (Tukey) comparing all groups, with an unpaired *t*-test (comparing two groups) or with a paired *t*-test (body mass before/after). At the time point of 21 days, no statistical comparison with the 160 MBq group was made due to too few blood samples. Statistical analysis was performed with GraphPad Prism (GraphPad Prism 9.3.0 for MacOS; GraphPad Software; GraphPad, Bethesda, MD). Values of *p* < 0.05 were considered significant and are marked in the figures (* *p* < 0.05, ** *p* < 0.01, *** *p* < 0.001, **** *p* < 0.0001).

## 3. Results

### 3.1. White Blood Cells

**Figure 1:**
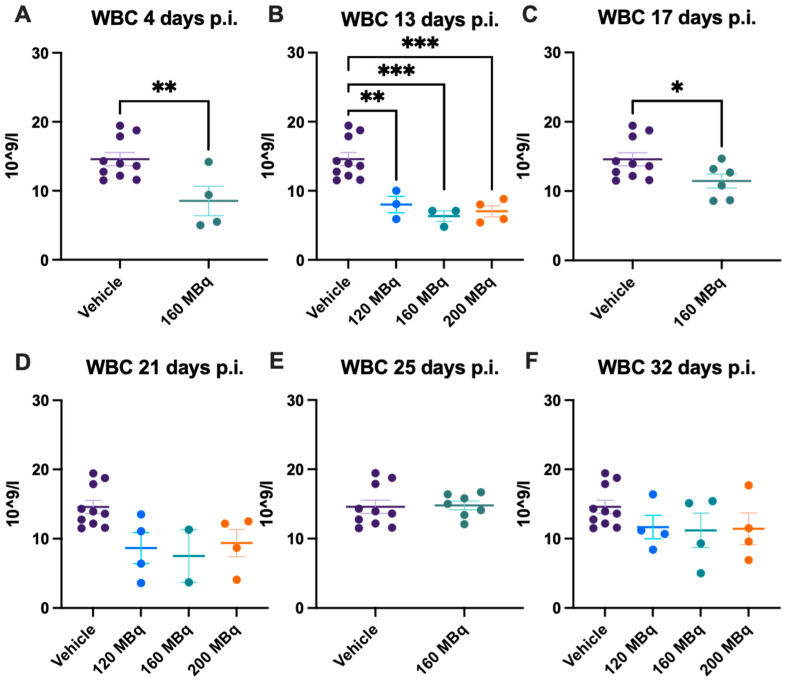
White blood cell (WBC) count after 4 (**A**), 13 (**B**), 17 (**C**), 21 (**D**), 25 (**E**), and 32 days (**F**) post injection of 120 MBq (**B**,**D**,**F**), 160 MBq (**A**–**F**), or 200 MBq (**B**,**D**,**F**). Values are presented as individual plots with group mean ± SEM. Differences between groups were analyzed using an unpaired *t*-test (**A**,**C**,**E**) or one-way ANOVA with post-hoc Tukey (**B**,**D**,**F**). * *p* < 0.05, ** *p* < 0.01, *** *p* < 0.001.

**Figure 2:**
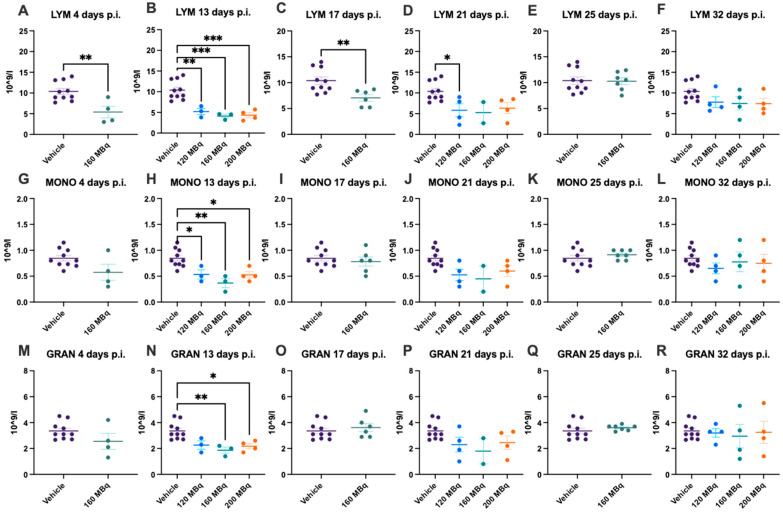
Lymphocyte (LYM) count after 4 (**A**), 13 (**B**), 17 (**C**), 21 (**D**), 25 (**E**), and 32 days (**F**) post injection of 120 MBq (**B**,**D**,**F**), 160 MBq (**A**–**F**), or 200 MBq (**B**,**D**,**F**). Monocyte (MONO) count after 4 (**G**), 13 (**H**), 17 (**I**), 21 (**J**), 25 (**K**), and 32 days (**L**) post injection of 120 MBq (**H**,**J**,**L**), 160 MBq (**G**–**L**), or 200 MBq (**H**,**J**,**L**). Granulocyte (GRAN) count after 4 (**M**), 13 (**N**), 17 (**O**), 21 (**P**), 25 (**Q**), and 32 days (**R**) post injection of 120 MBq (**N**,**P**,**R**), 160 MBq (**M**–**R**), or 200 MBq (**N**,**P**,**R**). Values are presented as individual plots with group mean ± SEM. Differences between groups were analyzed using an unpaired *t*-test (**A**,**C**,**E**,**G**,**I**,**K**,**M**,**O**,**Q**) or one-way ANOVA with post-hoc Tukey (**B**,**D**,**F**,**H**,**J**,**L**,**N**,**P**,**R**). * *p* < 0.05, ** *p* < 0.01, *** *p* < 0.001.

There were no significant differences in blood values in control animals at the different time points during the experiment; hence, data for all control animals (a mean from the different time points of each individual animal) are presented at all timepoints. Already after 4 days post injection of 160 MBq ^177^Lu-PSMA-617, there was a significant lowering of WBC (Figure 1A), notably in lymphocytes but not in monocytes and granulocytes (Figure 2A,G,M). The drop in WBC was significant in all groups receiving activity, 120, 160, and 200 MBq, after 13 days (Figure 1B), where monocytes and granulocytes, in addition to lymphocytes (with an exception for 120 MBq), also were significantly lower than the control group (Figure 2B,H,N).

After 17 days, the 160 MBq group still had lower WBC (Figure 1C) and lymphocytes (Figure 2C), but the monocytes and granulocytes had recovered to a similar level as control animals (Figure 2I,O). At 21, 25, and 32 days, there were no significant differences between control and animals receiving ^177^Lu-PSMA-617 (Figure 1D–F and Figure 2J–L,P–R), suggesting no long-lasting hematotoxicity. Overall, there was no correlation between activity received and decrease in white blood cells at the different time points, not when correcting for body mass differences either (Supplementary Figure S1).

### 3.2. Red Blood Cells

**Figure 3:**
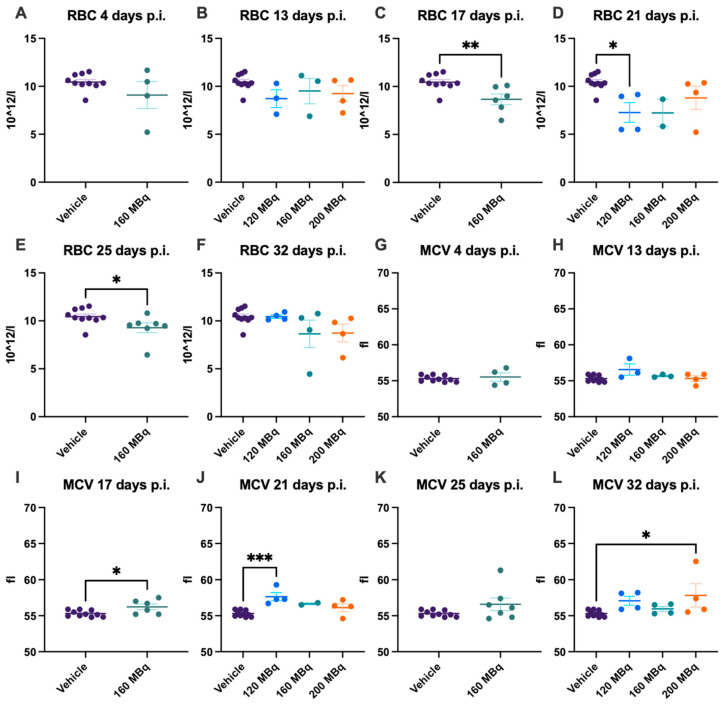
RBC count after 4 (**A**), 13 (**B**), 17 (**C**), 21 (**D**), 25 (**E**), and 32 days (**F**) post injection of 120 MBq (**B**,**D**,**F**), 160 MBq (**A**–**F**), or 200 MBq (**B**,**D**,**F**). MCV after 4 (**G**), 13 (**H**), 17 (**I**), 21 (**J**), 25 (**K**), and 32 days (**L**) post injection of 120 MBq (**H**,**J**,**L**), 160 MBq (**G**–**L**), or 200 MBq (**H**,**J**,**L**). Values are presented as individual plots with group mean ± SEM. Differences between groups were analyzed using an unpaired *t*-test (**A**,**C**,**E**,**G**,**I**,**K**) or one-way ANOVA with post-hoc Tukey (**B**,**D**,**F**,**H**,**J**,**L**). * *p* < 0.05, ** *p* < 0.01, *** *p* < 0.001.

**Figure 4:**
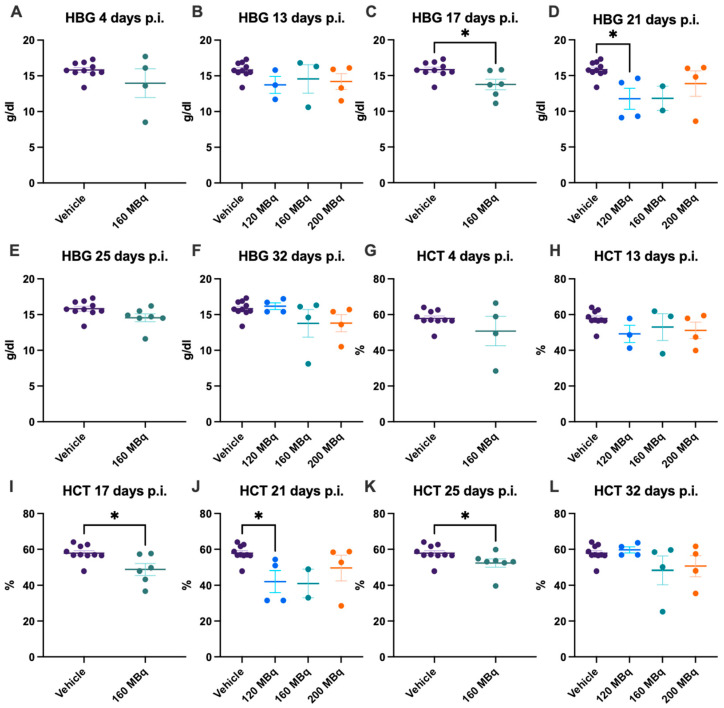
HBG after 4 (**A**), 13 (**B**), 17 (**C**), 21 (**D**), 25 (**E**), and 32 days (**F**) post injection of 120 MBq (**B**,**D**,**F**), 160 MBq (**A**–**F**), or 200 MBq (**B**,**D**,**F**). HCT after 4 (**G**), 13 (**H**), 17 (**I**), 21 (**J**), 25 (**K**), and 32 days (**L**) post injection of 120 MBq (**H**,**J**,**L**), 160 MBq (**G**–**L**), or 200 MBq (**H**,**J**,**L**). Values are presented as individual plots with group mean ± SEM. Differences between groups were analyzed using an unpaired *t*-test (**A**,**C**,**E**,**G**,**I**,**K**) or one-way ANOVA with post-hoc Tukey (**B**,**D**,**F**,**H**,**J**,**L**). * *p* < 0.05.

At the earliest time point investigated, 4 days post injection, the 160 MBq group showed no significant difference in the number of RBCs, difference in size, estimated by mean cell volume (MCV), level of HBG, or hematocrit (HCT) compared to control animals (Figure 3A,G and Figure 4A,G). No significant difference in any of these parameters could be seen when comparing all groups, 120, 160, and 200 MBq, with controls at 13 days post injections, except that the spread was larger in the treated groups (Figure 3B,H and Figure 4B,H). After 17 days, a lower number of RBCs and an increase in MCV were evident in the 160 MBq group (Figure 3C,I), indicating mild anemia resulting from the radiation. A lower level of HBG and a decrease in HCT were also apparent in this group at this time point (Figure 4C,I).

After 21 days, all groups showed a tendency toward a lower number of RBCs, although only significant in the 120 MBq group (Figure 3D). However, when analyzing the blood at 25 days post injection, the number of RBCs and HCT were again significantly lower in the 160 MBq animals (Figure 3E and Figure 4K). After 32 days, all studied parameters involving RBCs were normalized and did not differ from control mice, with the only exception of MCV in the 200 MBq group (Figure 3L). Similar to the RBCs, the spread, in general, was larger in the treated groups for MCV, HBG, and HCT than in the control group (Figure 3G–L and Figure 4). Further, as with the WBC, the decrease in RBCs and HBG levels did not have a correlation to injected activity, and no significant differences between the 120, 160, and 200 MBq were seen at any time point.

### 3.3. Platelets

**Figure 5:**
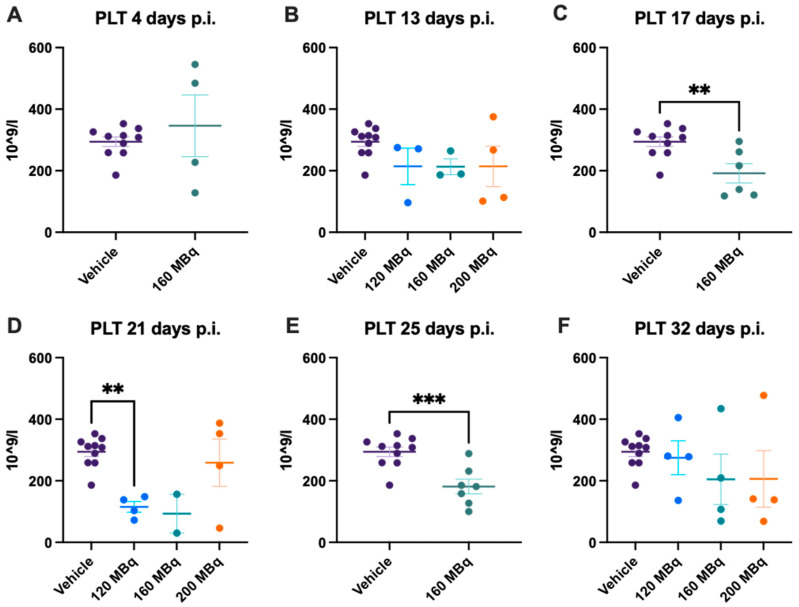
Platelet count after 4 (**A**), 13 (**B**), 17 (**C**), 21 (**D**), 25 (**E**), and 32 days post injection of 120 MBq (**B**,**D**,**F**), 160 MBq (**A**–**F**), or 200 MBq (**B**,**D**,**F**). Values are presented as individual plots with group mean ± SEM. Differences between groups were analyzed using an unpaired *t*-test (**A**,**C**,**E**) or one-way ANOVA with post-hoc Tukey (**B**,**D**,**F**). ** *p* < 0.01, *** *p* < 0.001.

After 17 days, a significant drop in platelet levels could be seen in the 160 MBq group, which remained significantly lower compared to the control group after 21 days (Figure 5C,D). This was also seen for the 120 MBq group, but not in the 200 MBq group, although there was a large spread in the latter group. The reduction in platelet count was still significant after 25 days in the 160 MBq group. At the last time point, 32 days, animals that had received activity had a large interval in the number of platelets, but at the group level, there was no difference compared to the control group.

### 3.4. Body Mass and Health Assessment

**Figure 6:**
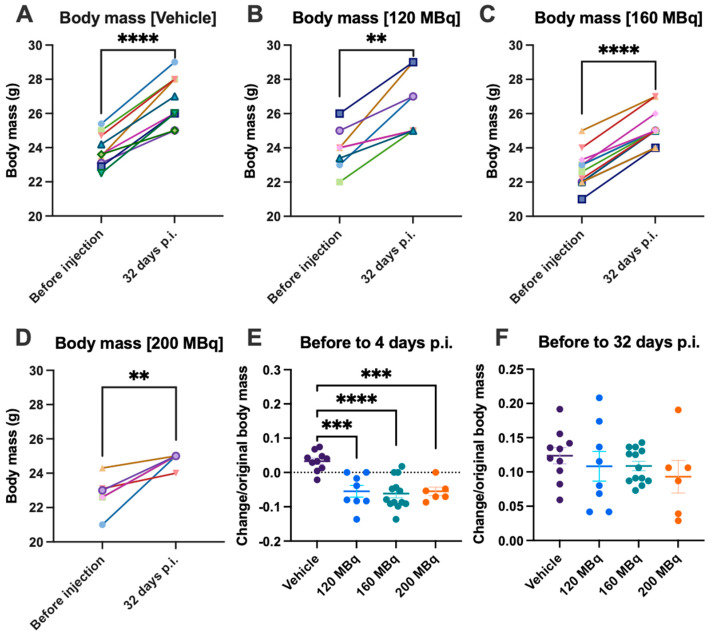
Body mass gain from before injections until 32 days post injections for (**A**) vehicle, (**B**) 120 MBq, (**C**) 160 MBq, and (**D**) 200 MBq. The increase or decrease in body mass presented as a fraction of original body mass with group mean ± SEM after 4 days (**E**) and 32 days post injections (**F**). Differences between before and after (**A**–**D**) were analyzed with a paired *t*-test, and differences between groups (**E**,**F**) were analyzed using one-way ANOVA with post-hoc Tukey. ** *p* < 0.01, *** *p* < 0.001, **** *p* < 0.0001.

Body mass was continuously monitored to assess health. Although mice receiving activity had an initial significant body mass loss (presented as gained/lost fraction from starting body mass) (Figure 6E), all mice had gained additional body mass at the end of the experiment (Figure 6A–D,F). There was no significant difference in the body mass gains between groups (presented as gained mass relative to starting body mass).

Only one mouse was sacrificed before the end of the experiment, belonging to the 160 MBq group. However, this was not due to radiation-induced damage. No other adverse effects, such as bleedings, were apparent during the course of the experiments.

## 4. Discussion

In patients with mCRPC, ^177^Lu-PSMA-617 RLT has shown promising results but is not curative today. Increased activity and/or more fractions or combining RLT with other therapies, such as radioprotectors, could improve the outcome for patients. To study this, accurate animal models are needed. One major possible adverse effect of injecting higher than normal amounts of activity in RLT, up to 120 MBq, has routinely been used [12,19] is the impact on the hematological profile, i.e., the risk of hematotoxicity, of the studied mouse models. In this work, we have shown that injection of ^177^Lu-PSMA-617 at activities of 120, 160, and 200 MBq results in a significant reduction in WBC, RBC, and platelets in addition to an initial body mass loss in mice. However, blood values were normalized within 32 days, and final body mass gain occurred to the same degree as in control mice. Therefore, although prolonged hematotoxicity is not a concern in this mouse model of ^177^Lu-PSMA-617, the initial drop in blood cell counts and body mass loss should be taken into account. Liver, kidney, and prolonged bone marrow toxicity were not investigated and could, in a longer time span, interfere with the health of animals.

This study focuses on the effects on the bone marrow from ^177^Lu-PSMA-617, but it is also very important to ensure that the injected solution does not include any free ^177^Lu, which has been shown to primarily distribute to the bone in rodents [21] and give a large, absorbed dose to the bone marrow. Hence, the radiochemical purity of the radioligand in our study was above 99% to minimize the risk of any free ^177^Lu increasing the absorbed bone marrow dose.

The data revealed an early drop in WBC (in all subtypes), with the estimated nadir around day 13 p.i. (Supplementary Figure S2) which is similar to the nadir reported for ^177^Lu labeled antibodies [22,23]. The animals never reached critical levels of WBC depletion but had approximately 50% reduction as the lowest level (13 days). The RBC and platelets reached the nadir later, around 21 days p.i. The lowered levels of platelets were not associated with adverse effects such as bleeding in our study. It should also be noted that animals receiving activity presented a larger variation in numbers of WBC, RBC, and especially platelets compared to control animals, which suggests that there is an individual variation in reaction to the radioactivity even though the animals are a homogeneous group. The recovery within a month was similar to the previously reported 4 weeks after 120 MBq [19]; hence, higher activities, up to 200 MBq, do not increase the time to recovery.

A drop in WBC correlated to absorbed dose to the bone marrow has been detected earlier with ^177^Lu [24,25]. However, here in our study, we did not find any such correlation between absorbed dose and WBC, even after adjusting for activity and body mass. This might be due to the faster clearance of the ^177^Lu-PSMA-617 than, for example, ^177^Lu attached to antibodies, and therefore a relatively low absorbed dose to the bone marrow. A suitable indicator is estimations from blood. In the biodistribution data from Fendler et al., blood had low levels of prolonged retention of activity compared to other tissues investigated, with kidneys having the highest organ uptake of healthy tissues [19].

Bone marrow effects from radiation are related to the absorbed dose to the bone marrow. Directly measuring the uptake of activity in the bone marrow is difficult and inaccurate. It is possible, however, to make a rough estimation of the bone marrow absorbed dose using the measured activity in blood. Here, we used the published activity of ^177^Lu-PSMA-617 in blood in (female) BALB/c nude mice from Umbricht et al. in 2019 [26] and the calculation of Sgouros in 1993, that bone marrow uptake is 36% of blood activity [27] together with our own data to calculate the number of decays in bone marrow for each group (see method in supplementary material). If we assume that all electrons from ^177^Lu [28] are deposited locally, we get absorbed doses of 0.18, 0.23, and 0.29 Gy to the bone marrow for injected activities of 120, 160, and 200 MBq, respectively. If we instead use the bone marrow to bone marrow S-value published by Larsson et al. in 2011, we get 0.14, 0.18, and 0.23 Gy [29]. These are very rough estimations, and the 36% estimation was made for radiolabelled antibodies in humans and is probably not fully applicable to small molecules in mice. It should also be noted that the protein amount injected is 1.0 nmol per mouse in the blood uptake data [30] but we injected 1.9, 2.6, or 3.2 nmol. In Fendler et al. there was about a 20% difference, however not significant, in blood uptake at 4 days p.i. between injections of 1.0 and 1.9 nmol [19]. Less blood (and bone) uptake with increased ligand amount could help explain our observed lack of difference between injected activity levels. Therefore, even though the activity is considerably higher, measured in GBq/kg, than the activity given to patients (~5–8 GBq/cycle [20,31,32]), the absorbed dose in the bone marrow is still much lower than the generally accepted bone marrow absorbed dose in patients of 2 Gy [33,34]. In the VISION trial, an absorbed dose to the red marrow of 1.5 Gy was recently reported after 6-cycles [35]. It is not completely understood if bone marrow and kidney absorbed doses can be translated from humans to rodents in regards to biological response since higher maximum doses to, at least, bone marrow has been reported to be tolerable in rodents [25].

In our previous study, using the same animal model, we estimated an absorbed dose to the kidneys of 0.07 Gy per injected MBq of ^177^Lu-PSMA-617 [12]. Consequently, the animals in this study are expected to have received 8.4, 11.2, and 14 Gy to the kidneys for the injected activities of 120, 160, and 200 MBq. When animals received a single injection of 100 MBq ^177^Lu-PSMA-617, the blood values recovered after an initial drop [12]. The kidney absorbed dose was in line with or slightly lower than values reported in other studies [26,31,36]. This corresponds to, approximately, the absorbed dose given to patients after one fraction [31,32]. Higher administered activities, i.e., more fractions, lead to better tumor control in mice [19]. Similarly, tumor control in humans is affected by the number of fractions, with recommended 2–6 cycles depending on response, prognosis and risk factors [37]. The kidney absorbed doses in animal studies have been much lower compared to the absorbed dose received by patients in the clinic. Knowing that up to 200 MBq is well-tolerated in mice, this can be used to further study combination therapies or multiple fractions and thereafter evaluate kidney toxicity over an extended period of time. However, the bone marrow absorbed dose might still lack relevance when regarding RLT mouse models since, as reported here, the absorbed bone marrow dose is probably still very low and far from the absorbed dose in patients.

The choice of animal, BALB/cAnNRj mice, is based on the possibility of introducing tumors. However, our study is only applicable to non-tumor-bearing animals since the addition of a tumor would affect both the biodistribution of the radioligand and the overall health of the animal. It also only describes the hematotoxicity following one instance of treatment, and any study with multiple fractions would have to observe if recovery is impaired after the second fraction due to possible decreased bone marrow cell viability.

Our group has previously reported that the radical scavenger and heme-binding protein A1M protects kidney function in a mouse model of ^177^Lu-PSMA-617. Mice that did not receive co-injections with A1M had a faster deterioration of renal uptake and excretion [12]. Kidney toxicity was not examined in the present study, but the results presented here may allow for injecting animals with higher activities of fractionated therapy and, at the same time, studying the effect of A1M, or other radioprotectors, on reducing acute and late occurring renal damage. A1M has also been reported to have hemoprotective abilities with therapeutic opportunities in a wide range of diseases [38,39]. Therefore, in PRRT and RLT, a potential target for A1M could be to reduce the hematotoxicity, with the RBC being the most likely therapeutic target.

In conclusion, the hematotoxicity resulting from the infusion of radioactivity doses up to 200 MBq in our mouse model is transient, and mice exhibit normalized blood values within a month with no other apparent adverse effects (e.g., deaths, bleeding, or sustained body mass loss). These activities in animal models may increase the relevance when studying renal toxicity in animal models and, hence, help improve therapeutic translation from animal models to the clinic.

## Data Availability

Data will be available on request.

## Supplementary Materials

The following supporting information can be downloaded at: https://www.mdpi.com/article/10.3390/pharmaceutics14040731/s1, Figure S1: Correlation WBC count with MBq/g; Figure S2: WBC, RBC, and PLT levels over time; Figure S3: Data points for uptake in blood and fitted model for bone marrow absorbed dose.
