# Supplementary material for: Hematological Toxicity in Mice after High Activity Injections of 177Lu-PSMA-617
Source: Pharmaceutics. 2022 Mar 28;14(4):731. doi: 10.3390/pharmaceutics14040731 (PMC9032768; doi:10.3390/pharmaceutics14040731)
Supplement: Supplementary file 1 [file pharmaceutics-14-00731-s001.zip › pharmaceutics-1629344-supplementary.pdf]

pharmaceutics

Supplementary Materials: Hematological Toxicity in Mice after High Activity Injections of ^{177}Lu-PSMA-617

Amanda Kristiansson, Oskar Vilhelmsson Timmermand, Mohamed Altai, Joanna Strand, Sven-Erik Strand, Bo Åkerström and Anders Örbom

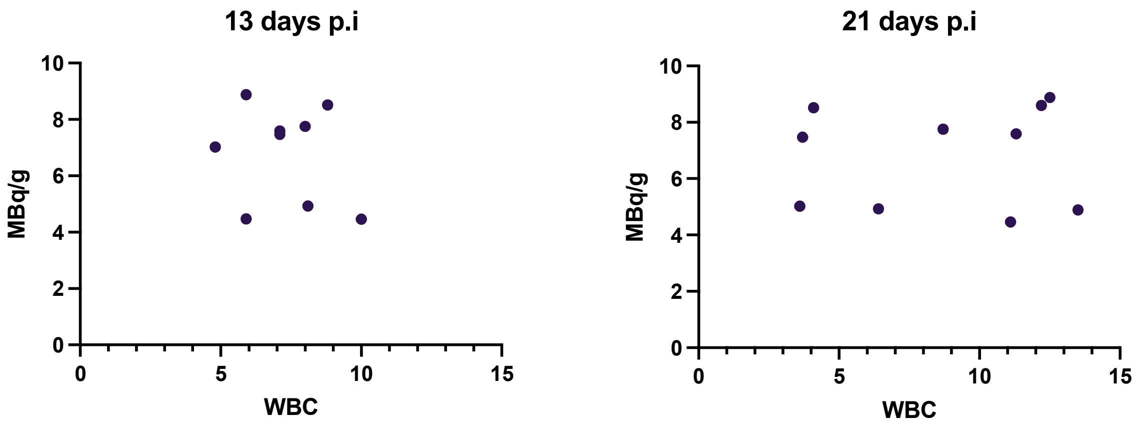

Figure S1. No correlation was seen between injected activity (MBq), after correction for body mass, and WBC 13 and 21 days p.i. The exact activity injected was divided by the body mass at time of the injection (MBq/g) and plotted against WBC (x 10^9).

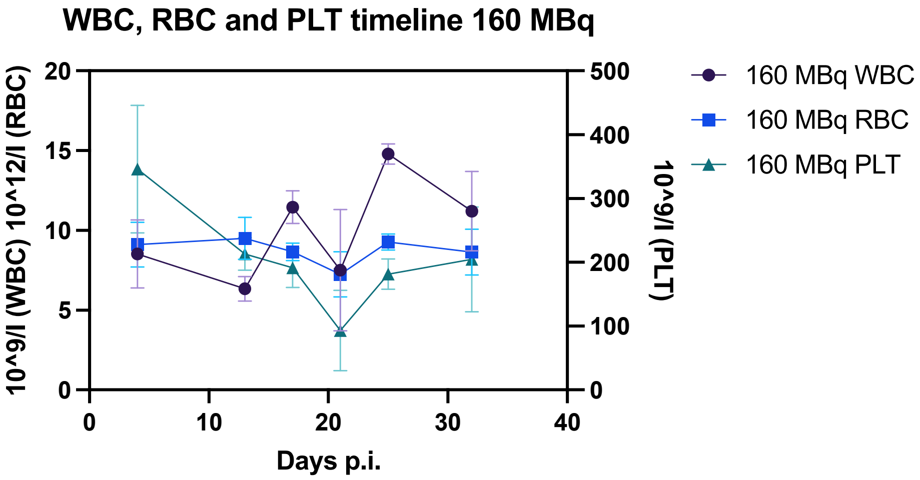

Figure S2. WBC, RBC and PLT levels after injections with 160 MBq. Data is presented over time from 4 to 32 days.

Estimation of decays in bone marrow

A bi-exponential function was fitted to the blood uptake data, and to an additional data point of 0 % IA/g uptake at 0 minutes p.i. The function described in Eq. (1) was fitted to the data using code written in Interactive Data Language version 8.5 (IDL; ITT Exelis; McLean, VA, USA).

$$A_0(f_1 \cdot e^{-\alpha_1 t} + f_2 \cdot e^{-\alpha_2 t}) \quad (1)$$

A Powell minimization function was used to find the values of A_0, f_1, α_1, f_2 and α_2 based on a least squares measure of the difference between data and calculated values. The variables, f_1, α_1 and α_2 were only allowed to be above zero, to avoid biologically unrealistic fits. The fitted model for the first 300 minutes post injection can be seen in supplementary figure 1. Once the variables were optimized, the function was plotted from t=0 to t=192 hours and then the physical decay of ^{177}Lu was applied and the function numerically integrated to yield the amounts of decay per gram per injected activity. It was found that the maximum number of decays was reached already at 225 minutes post injection. This number of decays for blood was multiplied by the 36% factor to serve as number of decays for bone marrow.

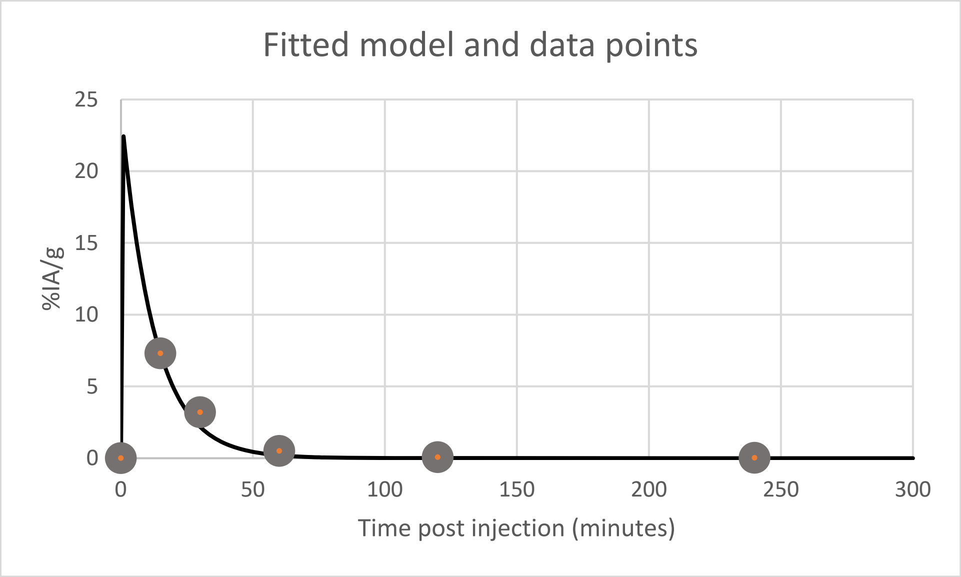

Figure S3. Data points for uptake in blood (gray dots) and fitted model (black line).
